# Toxoplasmosis Behind Bars: One Health Approach on Serosurvey Dynamics and Associated Risk Factors for Women Inmates, Correctional Officers, and In-Prison Feral Cats

**DOI:** 10.1155/2024/9390381

**Published:** 2024-02-27

**Authors:** Gabriel Luís Brucinski Pinto, Vamilton Alvares Santarém, Juliano Ribeiro, Roberto Teixeira de Souza Filho, Danilo Alves de França, Gustavo Nunes de Moraes, Jully Kosloski, Leandro Meneguelli Biondo, Rogério Giuffrida, Hélio Langoni, Louise Bach Kmetiuk, Alexander Welker Biondo

**Affiliations:** ^1^Graduate College of Cell and Molecular Biology, Federal University of Paraná (UFPR), Curitiba 80035-050, Brazil; ^2^Graduate College in Animal Sciences, University of Western São Paulo (UNOESTE), Presidente Prudente, SP 19050-920, Brazil; ^3^Department of Animal Production and Preventive Medicine, School of Veterinary Medicine and Animal Science, São Paulo State University (UNESP), Botucatu, SP 18618-681, Brazil; ^4^National Institute of the Atlantic Forest (INMA), Brazilian Ministry of Science Technology and Innovation, Santa Teresa, ES 29650-000, Brazil; ^5^Interdisciplinary Graduate Studies, University of British Columbia, Kelowna, BC V6T1Z4, Canada

## Abstract

Brazil holds the third highest general and fifth female incarcerated population worldwide. Despite the incarceration ecosystem that may favor the spreading of zoonotic diseases, particularly when unattended animals are present, no comprehensive study has focused on toxoplasmosis dynamics in such environment. Accordingly, the present study has aimed to serologically assess anti-*Toxoplasma gondii* (IgG) antibodies by indirect immunofluorescent antibody test in inmates, correctional officers, and feral cats at the Women's State Penitentiary of Parana, southern Brazil. In overall, 230/506 (45.5%; CI 95%: 41.2–49.8) incarcerated women, 31/91 (34.1%; 95% CI: 25.2–44.3) correctional officers, and 23/39 (59.0%; CI 95%: 43.2–72.9) cats were seropositive to anti-*T. gondii* antibodies. Logistic regression revealed that seropositivity likelihood increased with consumption of raw meat (*p*=0.040) and decreased with elementary educational level (*p*=0.001). No statistical difference was found comparing seropositivity between inmates and correctional officers (*p*=0.057). As women inmates have been considered among the most vulnerable groups in disease morbidity and mortality, seropositivity observed herein may be directly related to vulnerability and high *T. gondii* oocyst exposure dispersed in cat feces during incarceration.

## 1. Introduction

Brazil has been ranked as the third highest general incarcerated population worldwide, with 811,000 persons deprived of liberty, surpassed only by China with 1.69 and the USA with around 2.0 million people [1]. In addition, Brazil holds the fifth largest female incarcerated population with 37,380 individuals, only behind the USA (205,400), China (103,766), Russia (53,304), and Thailand (44,751) [2]. Despite being considered a small fraction of the total population worldwide, female incarceration has increased by 50% in the past 15 years [1, 3].

Incarcerated persons, along with refugees and homeless, have been considered the three most vulnerable populations in disease morbidity and mortality [4]. Incarceration environment may increase the likelihood of infectious diseases, as reclused populations may exacerbate vulnerability due to movement restriction, mutual convivence within confined spaces, and limited health assistance [5]. Such daily life within overcrowded prisons and their precariousness has made the environment conducive to disease spreading [6]. For instance, the incidence of tuberculosis (TB) in prisons may be 10 times greater than the general population [7]. Susceptibility to infectious diseases may be even higher in immunocompromised populations, with four to fivefold more likelihood for toxoplasmosis in HIV-positive inmates [8, 9]. Furthermore, women inmates may have poor access to health care [3, 10].

Considered a ubiquitous protozoan parasite, *Toxoplasma gondii* has caused toxoplasmosis, a zoonotic infection with a worldwide distribution [11]. The disease has a complex epidemiology, being transmitted by ingestion of oocysts shed into feces of definitive feline hosts and contaminate water, soil, and crops, and by consumption of intracellular cysts in undercooked meat from intermediate hosts [12]. Despite usually being an asymptomatic disease in immunocompetent individuals, toxoplasmosis has been considered relevant to public health primarily within the context of congenital toxoplasmosis or postnatally acquired disease in immunocompromised patients [13].

The complexity of health problems worldwide, particularly in developing countries, has demanded a more holistic and systemic approach involving interrelations of medical and environmental sciences, in a One Health convergence [14]. One in 1,000 Latin American persons may be affected by a parasitic zoonosis, according to the World Health Organization, with toxoplasmosis indicated as among the most frequent and priority for public actions [15].

Despite the closed ecosystem of incarceration that may favor the spreading of zoonotic diseases, particularly when unattended animals are present, no comprehensive study has focused on toxoplasmosis dynamics in the women inmate population. Accordingly, the present study has aimed to serologically assess anti-*T. gondii* (IgG) antibodies in all inmates, correctional officers, and feral cats at the Women's State Penitentiary of Parana, southern Brazil.

## 2. Materials and Methods

### 2.1. Ethical Aspects

The present study was performed in coordination with the administration of the Penitentiary Department of the Piraquara Complex, officially included as part of their official activities, along with COVID-19 sampling and testing.

### 2.2. Study Design

The present study was a cross-sectional serosurvey of anti-*T. gondii* antibodies (IgG) and associated risk factors in women deprived of liberty, correctional officers, and feral cats of a state penitentiary in southern Brazil. In addition, a longitudinal approach was performed to compare seroprevalence during the COVID-19 epidemics.

### 2.3. Study Population and Area

**Figure 1:**
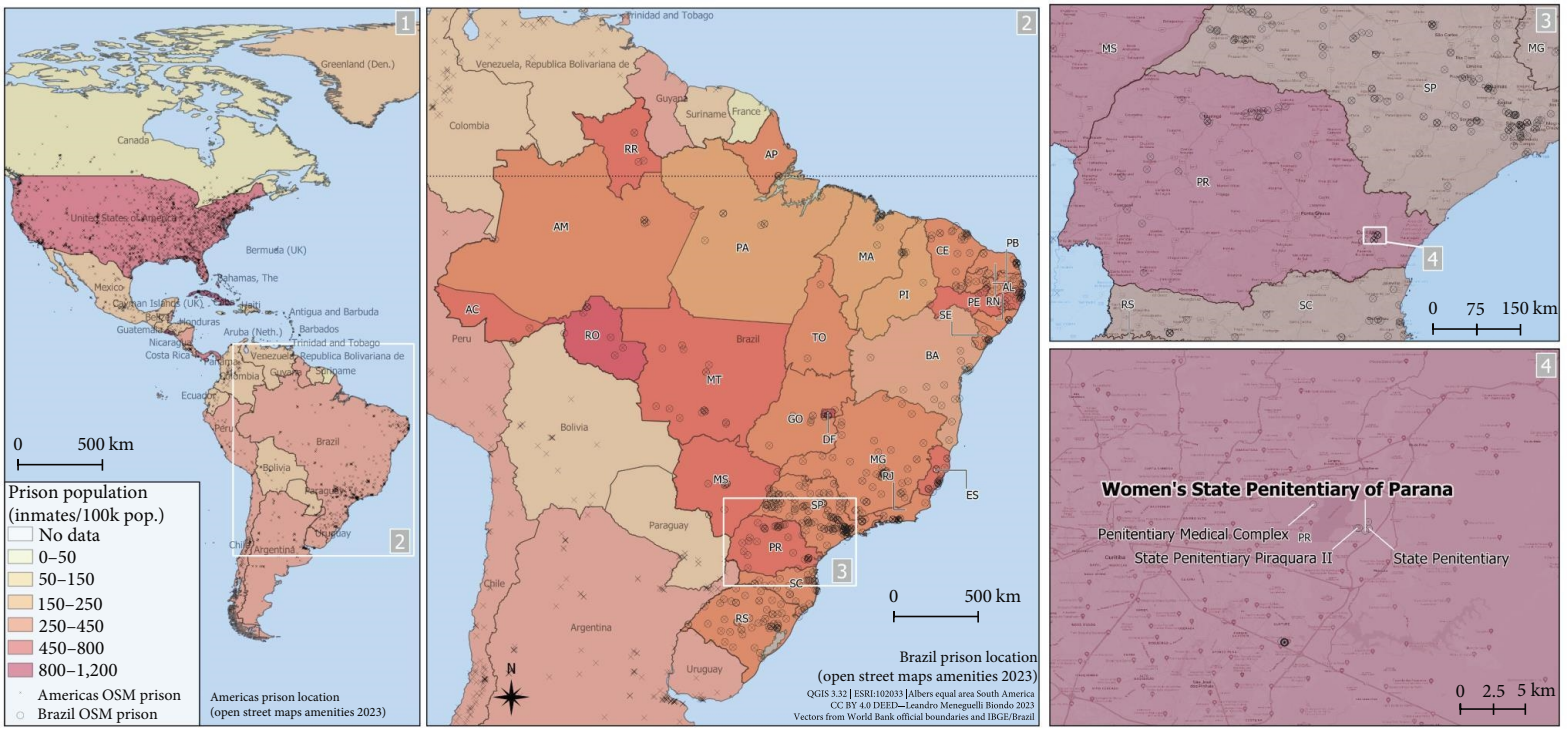
Sampling location of inmates, correctional officers, and feral cats in the Women's State Penitentiary of Parana, southern Brazil. Prison population distribution in Brazilian states was also highlighted, according to the Infopen system from the Public Ministry [16].

Both inmate and correctional officer populations were sampled in total twice, first in October 2020 and later in November 2021, while feral cats were captured and sampled during the study period. Socioepidemiological information was obtained by signed questionnaires. The study was fully conducted at the Women's State Penitentiary of Parana State (Figure 1), part of the Piraquara Correctional Complex, third in population in the Brazilian incarceration system with around 7,000 men and women inmates held by 800 correctional officers and arranged in seven different units at the time. The complex, located in the Piraquara municipality (25°24′59′′S, 49°04′46′′W), is part of the Curitiba metropolitan area, capital of Paraná State and the eighth largest Brazilian city with around 3.2 million habitants.

### 2.4. Blood Sampling and Epidemiological Data

Participants herein, including inmates and officers, were sampled after signing a consent form and filling out an epidemiological questionnaire. Approximately 8 mL of whole blood was collected by cephalic venipuncture in humans and by jugular venipuncture in cats, collected by certified nurses and veterinarians, respectively. Samples were placed in a tube with serum separator gel, centrifuged at 800 *g* for 5 min, and subsequently, serum separated and kept at −20°C until processing.

Epidemiological data were obtained from inmates and correctional officers after informed notice, confidentiality of their identities, and right to refuse participation at any time. Participants were asked to voluntary sign the Free and Informed Consent Term in compliance with the National Brazilian Health Council (resolution no. 441/2012). The epidemiological questionnaire was based on potential associated risk factors for toxoplasmosis (Table 1).

As counterpart and ethical research, the study herein included a free-of-charge assistance to feral cats at the penitentiary, conducted by the shelter medicine service at the Veterinary Teaching Hospital, Federal University of Paraná. Once per week, feral cats were trapped inside the buildings and courtyards, examined, treated (if necessary), dewormed, given anti-flea, vaccinated, microchipped, and taken for neutering/spaying. Kittens and docile cats were sent for adoption, while healthy feral cats were released back into the penitentiary and monitored.

### 2.5. Serological Testing

Serological testing of anti-*T. gondii* antibodies were performed by indirect immunofluorescent antibody test (IFAT) in both human and cat samples, using correspondent species-specific conjugates, as previously established [17]. Serial dilutions from 1 : 16 to 1 : 4,096 were applied in pH 7.2 phosphate-buffered saline solution, using a cutoff titer of ≥16 IU. Immunofluorescence slides were presensitized with 0.1% formaldehyde to inactivate *T. gondii* tachyzoites (RH strain), obtained from an intraperitoneal lavage in Swiss mice following 3-day inoculation.

### 2.6. Statistical Analyses

All statistical analyses were performed using R software. First, collected data were assessed for detecting missing values, outliers, and inconsistencies. Then, data were cleaned by excluding values based on the missingness proportion (>10%) and the nature of missing values. Continuous variables were transformed into binary indicators or categories. Descriptive analyses were performed to summarize the data using contingency tables.

Univariate analyses were performed to assess the relationship between each independent variable and seropositivity, applying either the chi-square or Fisher's exact test. All calculations were performed independently by group, with the inclusion of information given by inmates (506) and correctional officers (91). Dependent variables were selected for inclusion in the logistic regression model based on the significance of univariate analysis (*p*  < 0.2). Multicollinearity among independent variables was checked to avoid high intrinsic correlation. Odds ratio and confidence intervals were calculated to quantify the strength of associations in the logistic regression model.

The logistic regression model was refined by adding or removing variables based on their statistical significance, effect size, and theoretical relevance. This refinement process continued until a final model with parsimonious and interpretable results was obtained. The *T. gondii* antibody dynamics were compared based on inmates (133) tested in both samplings.

The area under the ROC curve (AUC) was computed as a measure of the model's discriminative ability, ranging from 0.5 (no) to 1 (perfect) discrimination. Confidence intervals for the AUC were estimated to assess the statistical significance of the discriminatory power.

A significant level of 5% was adopted for all statistical tests.

## 3. Results

### 3.1. Incarcerated Women

A total of 506 incarcerated women were included in the study, with ages ranging from 18 to 62 years (median: 32), mostly self-declared as white (269/506; 53.2%), having elementary education (337/506; 66.6%), and at least one child (412/506; 81.4%). Two women referred to self-pregnancy at the time of questionnaire application.

Overall, 230/506 (45.5%; CI 95%: 41.2–49.8) incarcerated women were seropositive to anti-*T. gondii* antibodies. Out of the inmates tested again after a 1-year interval, 92/133 (69.2%) presented the same results, while 12/133 (9.0%) became seronegative and 29/133 (21.8%) seropositive (Table 2). A statistically significant difference in proportions was found in seropositive results when comparing the two serological tests (McNemar's *chi*-squared = 6.24, df = 1, *p*-value = 0.012).

Risk factors associated to seropositivity for anti-*T*. gondii antibodies were assessed and presented (Table 3). Logistic regression revealed that seropositivity likelihood increased with consumption of raw meat (OR: 1.53; *p*=0.040) and decreased with elementary educational level (OR: 0.47; *p*=0.001).

Univariate analysis showed a significant proportion of seropositive women in the non-white group (*p*=0.036), but the variable was not retained in the logistic regression (*p*=0.052). Hours in cell and frequency in solarium were also fitted to be included in the logistic regression, but neither variable was retained in the final model.

All other assessed variables (age, cat owner before detention, contact with cats during detention, washing hands before meals, pregnant status, mother, historic of miscarriage, contact with soil during detention, biting nails) were not associated to seropositivity (*p*  > 0.05).

### 3.2. Correctional Officers

The group of correctional officers herein was constituted of 91 individuals aging from 21 to 63 (median: 44), mostly female (84/91; 92.3%), self-declared white (60/91; 65.9%), and with higher education (65/91; 71.4%).

Serological examination revealed 31/91 (34.1%; 95% CI: 25.2–44.3) seropositive officers for toxoplasmosis. No tested variable, including gender, ethnicity, educational level, contact with cats in the penitentiary, meals provided by the penitentiary, consumption of water in the penitentiary, historic of raw meat ingestion, and history of miscarriage, was associated to seropositivity (Table 4).

In addition, no statistical difference was found comparing seropositivity proportions between inmates and correctional officers (OR: 1.6; 95% CI: 1.010–2.575; *p*=0.0572).

### 3.3. Feral Cats

Out of the feral cats trapped in this study, 19/39 (48.7%) were males and 20/39 (51.3%) females, mostly adults. Overall, 23/39 (59.0%; CI 95%: 43.2–72.9) cats were seropositive for anti-*T. gondii* antibodies. No statistical difference (chi-square: 0.037; *p*=0.848) in seropositivity was verified considering cat age and gender.

## 4. Discussion

The present study represents the first serosurvey conducted to assess seroprevalence for toxoplasmosis in the female inmate population of Brazil. Previous studies worldwide have mostly focused on incarcerated pregnant women, revealing South America as the highest pooled metanalysis seroprevalence (56.2%; 50.5%–62.8%) for latent toxoplasmosis [18], similar to that observed in the general female population herein (45.5%). Even so, seroprevalence herein was higher than the male inmate population, with 43/170 (25.3%) in Malaysia [9] and 207/497 (41.6%) in Indonesia [8]. Such results should be carefully compared due to different cultural, socioeconomic, gender, and pathogen exposure found in various countries and inmate populations. Additionally, the antibody testing method and cutoff point adopted herein may also be associated with these differences.

Toxoplasmosis has been considered widely prevalent in Brazil [19], with the highest seroprevalence recorded to date revealed in a blood donor serosurvey (75.0%; CI 95%: 68.0–82.0) [20]. In addition, this neglected parasitic disease has been associated with poverty [21, 22], with a significantly higher prevalence of latent toxoplasmosis in pregnant women in low-income countries with low human development indexes [18]. Socioeconomic vulnerability has been associated to disease in Brazil [23] and is considered a contributing factor to the persistent occurrence of congenital toxoplasmosis [24]. The logistic analysis herein revealed that seropositivity was significantly reduced in inmates with high school, in agreement with low education as a risk factor for toxoplasmosis in puerperal women [25–27].

Ethnicity has been associated to toxoplasmosis in pregnant women, as observed in Brazil [28] and in the USA [22]. Although the univariate analysis herein has pointed out a statistically higher proportion of seropositive women in the non-white group, such a variable was not retained by logistic regression (*p*=0.052). Thus, future studies should be conducted with higher in mate samplings, if possible, in a multicentric design, to fully establish such ethnicity as an associated risk factor in female inmates in Brazil and abroad.

Although only two inmates in the present study self-declared pregnant at the time, most of them referred to having at least one child. Despite no statistical significance was found between seropositivity and motherhood or miscarriage, the last has been reported as a consequence of toxoplasmosis in pregnant women [29, 30]. As miscarriage may be a result of a variety of disorders, a higher sampling with a survey of concomitant causes should be performed to pinpoint the role of toxoplasmosis, alone or combined.

As expected, ingestion of raw meat increased the odds of seropositivity in the inmates herein (OR: 1.53), a major risk factor for toxoplasmosis worldwide [19, 31, 32]. As the majority of female prisons in Brazil have been supplied with ultra-processed food, mostly fried chicken (79.2%) as a meat source [33], food during incarceration should not be an important source of *T. gondii* infection. Similarly, such “fast-food effect” due to diet habits of mostly processed food has also been shown in homeless [34] and animal hoarding [35] populations in Brazil, which also presented lower anti-*T. gondii* seroprevalence than the general population. Meat processing and cooking were also shown to reduce *T. gondii* levels in meat products in the USA [36]. As human anti-*T. gondii* antibodies may provide a lifelong protective immunity [36], and infection via raw meat may have been acquired previously to incarceration.

Unwashed fruits and vegetables and untreated water may also be important routes of *T. gondii* transmission to women [12]. Although water and foodborne (unwashed fruits and vegetables) transmission have been reportedly among the most frequent routes of toxoplasmosis outbreaks in Brazil [36, 37], food distributed herein came from industrial kitchens commercially contracted outside the penitentiary (under tight inspection and food handling practices), as the in-prison kitchen was closed at the beginning of COVID-19 pandemics. Thus, unless contamination occurred during in-prison storage and handling, food and water consumed by inmates herein may be unlikely sources of infection during the survey.

The central point in the present study was the overlapping presence of 23/39 (59.0%) seropositive feral cats in the penitentiary, serving as an alert for the environmental source of *T. gondii* infection during incarceration. The seropositivity observed in feral cats herein was significantly higher than the 16.3% (46/282) observed in owned cats in the same city [38] but lower than the 84.4% (49/58) found in other Paraná state areas. In addition, 21.92% (98/447) of domestic cats were seropositive in northern Brazil, with a statistically significant association between age and serology among cats over 1 year old [39]. Contact with cats or with soil containing infective *T. gondii* oocysts has already been established as a risk factor for toxoplasmosis [14]. However, no association was observed herein between seropositivity and cat or soil contact, probably due to the high number of women who declared no contact with cats or with soil during the prison period. Nonetheless, penitentiary outdoor sunlight areas, daily used by female inmates, were heavily contaminated with *Toxocara cati* eggs [40], suggesting daily inmate close contact with cat feces.

As the feral cat population herein was not fully neutered and spayed at the beginning of samplings, undesirable cat litters may have been responsible for historical *T. gondii* oocyst shedding, transmission, and persistence. In addition, cats freely transit through inside and outside prison may have allowed contact with other sources of *T. gondii*, such as prey meat and contaminated water. Finally, the feral cat population herein has been maintained by food scraps of daily meals produced in prison prior to COVID-19, suggesting a potential sharing of infection sources.

Another strength of this study was to investigate the *T. gondii* seropositivity dynamics in a 1-year period. A fluctuation was observed in both seronegative and seropositive results, with a significant difference in the proportions of seropositive results comparing the two serological time tests. As tests were performed in duplicate, such findings herein may be attributed to IFAT sensitivity in general, which has ranged from 80.4% to 100%, and specificity from 91.4% to 95.8%, depending on the tested species [41]. Such findings could be attributed to IFAT sensitivity, but tests were performed in duplicate. Further, concurrent infection could lead to a disruption in immunocompetence and recrudescence of toxoplasmosis [42], with a positive correlation between serum anti-*T. gondii* IgG levels and in TB patients, for instance, as observed in Egypt [43]. In addition, an increase in TB has been associated to HIV in prison inmates in southern Brazil [44]. As information regarding HIV results were lost over 50%, no analysis was possible to draw between toxoplasmosis and HIV coinfection. Thus, further investigation should be conducted to evaluate the reciprocal influence of antibody dynamics for toxoplasmosis and concurrent infections.

The seroprevalence of correctional officers and potential associated risk factors for toxoplasmosis were also evaluated. The observed herein 34.1% seroprevalence was very close to the overall 35.0% observed in a global metanalysis [45]. No tested variable was associated to seropositivity, and seropositivity proportions between inmates and correctional officers were not associated.

As limitations of this study, although feral cat prevalence found herein has indicated high cat exposure and a high degree of environmental contamination, no *T. gondii* oocyst recovery from soil, environment contamination, and genotyping analysis were performed, and should be further investigated. This finding needs to be interpreted with caution due to the self-declared information given by inmates, which could lead to a bias and misinterpretation of the results. In addition, the combined use of another serological method (e.g., ELISA) with IFAT could help to better understand the seroconversion dynamics observed herein. According to a meta-analysis, the use of enzyme-linked fluorescent immunoassay, consisting of an immunoenzymatic method with the final fluorescence detection, has provided a 92% sensitivity and 80% specificity for the IgG marker, highly improving the sensitivity accuracy [46].

Finally, as women deprived of liberty have been considered among the most vulnerable groups in disease morbidity and mortality [4], seropositivity observed herein may be directly related to vulnerability and high *T. gondii* oocyst exposure dispersed in cat feces during incarceration.

## 5. Conclusion

The study herein represented the first serosurvey conducted to assess seroprevalence for toxoplasmosis in the female inmate population of Brazil. The seropositivity observed herein may be directly related to vulnerability and high *T. gondii* oocyst exposure dispersed in cat feces during incarceration. In addition, as the feral cat population herein was not fully neutered and spayed at the beginning of samplings, undesirable cat litters may have been responsible for historical *T. gondii* oocyst shedding, transmission, and persistence. Finally, cats freely transit through inside and outside the prison and may have allowed contact with different sources of *T. gondii*, such as water and prey.

## Figures and Tables

**Table 1:** Information for assessing the potential exposure and associated risk factors for toxoplasmosis in women inmates and correctional agents in a penitentiary of Paraná State, southern Brazil.

Topics	Gathered information	
	Inmates	Correctional agents
Socioeconomic characteristics	Age, ethnicity, educational level	Gender, age, ethnicity, educational level
Gestational characteristics	Pregnant, mother, history of miscarriage	History of miscarriage
Practices and hygienic habits in penitentiary	Hours in cell, contact with cats, frequency in solarium, washing hands before meals, contact with soil, biting nails	Contact with cats, meals provided by the penitentiary, consuming of water in the penitentiary
History	Cat owner before detention, raw meat ingestion	Raw meat ingestion

**Table 2:** Serological results by indirect immunofluorescent antibody test (IFAT) in a 1-year interval for anti-*Toxoplasma gondii* antibodies among incarcerated women (*N* = 133) in a penitentiary of Paraná State, southern Brazil.

IFAT results	Positive (%)	Negative (%)	Total
First testing	58 (43.6)	75 (56.4)	133
Second testing (1-year interval)	75 (56.4)	58 (43.6)	133

**Table 3:** Associated risk factors for anti-*Toxoplasma gondii* antibodies in incarcerated women (*N* = 506) in a penitentiary of Paraná State, southern Brazil.

	Serological results (%)		Univariate analysis		Multivariate analysis	
	Positive	Negative	OR (95% CI)	*p*	OR (95% CI)	*p*
	230 (45.5)	276 (54.5)	—	—	—	—
Age (years old)	—	—	—	0.301	—	—
18–26	55 (24.0)	68 (24.6)	1 (Reference)	—	—	—
27–31	58 (25.3)	65 (23.6)	1.10 (0.67–1.83)	—	—	—
32–38	45 (19.7)	72 (26.1)	0.77 (0.46–1.30)	—	—	—
>38	71 (31.0)	71 (25.7)	1.23 (0.76–2.01)	—	—	—
Ethnicity	—	—	—	0.036	—	—
Non-white	119 (52.0)	116 (42.2)	1 (Reference)	—	—	—
White	110 (48.0)	159 (57.8)	0.68 (0.47–0.96)	—	0.68 (0.45–1.00)	0.052
Educational level	—	—	—	0.001	—	—
Elementary	172 (74.8)	165 (60.0)	1 (Reference)	—	—	—
High school	44 (19.1)	93 (33.8)	0.46 (0.30–0.69)	—	0.47 (0.29–0.75)	0.001
Graduate	14 (6.09)	17 (6.18)	0.79 (0.37–1.67)	—	0.76 (0.32–1.71)	0.500
Hours in cell	—	—	—	0.007	—	—
All day	168 (75.7)	170 (63.9)	1 (Reference)	—	—	—
18 hr	54 (24.3)	96 (36.1)	0.57 (0.38–0.85)	—	0.95 (0.57–1.58)	0.841
Cat owner before detention	—	—	—	0.354	—	—
No	142 (66.0)	188 (70.4)	1 (Reference)	—	—	—
Yes	73 (34.0)	79 (29.6)	1.22 (0.83–1.80)	—	—	—
Contact with cats during detention	—	—	—	0.982	—	—
No	156 (72.9)	186 (72.4)	1 (Reference)	—	—	—
Yes	58 (27.1)	71 (27.6)	0.97 (0.65–1.46)	—	—	—
Frequency in solarium (per week)	—	—	—	0.067	—	—
No access	37 (17.6)	47 (18.7)	1 (Reference)	—	—	—
Once	64 (30.5)	100 (39.7)	0.81 (0.48–1.39)	—	0.89 (0.49–1.59)	0.687
Twice or more	109 (51.9)	105 (41.7)	1.32 (0.79–2.20)	—	1.40 (0.80–2.45)	0.235
Washing hands before meals	—	—	—	0.363	—	—
No	7 (3.08)	4 (1.48)	1 (Reference)	—	—	—
Yes	220 (96.9)	267 (98.5)	0.48 (0.12–1.65)	—	—	—
History of raw meat ingestion	—	—	—	0.021	—	—
No	127 (58.8)	183 (69.3)	1 (Reference)	—	—	—
Yes	89 (41.2)	81 (30.7)	1.58 (1.09–2.31)	—	1.53 (1.02–2.29)	0.040
Pregnant	—	—	—	1	—	—
No	216 (99.5)	268 (99.6)	1 (Reference)	—	—	—
Yes	1 (0.46)	1 (0.37)	1.24 (0.03–48.6)	—	—	—
Mother	—	—	—	0.364	—	—
No	34 (15.0)	50 (18.5)	1 (Reference)	—	—	—
Yes	192 (85.0)	220 (81.5)	1.28 (0.80–2.08)	—	—	—
History of miscarriage	—	—	—	0.545	—	—
No	144 (69.2)	168 (66.1)	1 (Reference)	—	—	—
Yes	64 (30.8)	86 (33.9)	0.87 (0.58–1.29)	—	—	—
Contact with soil during detention	—	—	—	0.791	—	—
No	207 (93.7)	252 (92.6)	1 (Reference)	—	—	—
Yes	14 (6.33)	20 (7.35)	0.86 (0.41–1.73)	—	—	—
Biting nails	—	—	—	0.485	—	—
No	151 (68.9)	173 (65.5)	1 (Reference)	—	—	—
Yes	68 (31.1)	91 (34.5)	0.86 (0.58–1.26)	—	—	—

**Table 4:** Associated risk factors for anti-*Toxoplasma gondii* antibodies in correctional officers (*N* = 91) in a penitentiary of Paraná, southern Brazil.

	Serological results		Univariate analysis	
Variables	Positive (%)	Negative (%)	OR (95% CI)	*p* Overall
	31 (34.1)	60 (65.9)	—	—
Gender	—	—	—	0.224
Female	27 (87.1)	57 (95.0)	1 (Reference)	—
Male	4 (12.9)	3 (5.00)	2.75 (0.54–15.8)	—
Age (years old)	—	—	—	0.204
<38	9 (30.0)	12 (20.3)	1 (Reference)	—
38–43	6 (20.0)	15 (25.4)	0.54 (0.14–1.98)	—
44–50	4 (13.3)	18 (30.5)	0.31 (0.07–1.21)	—
>50	11 (36.7)	14 (23.7)	1.05 (0.32–3.48)	—
Ethnicity	—	—	—	0.695
Non-white	9 (29.0)	21 (35.6)	1 (Reference)	—
White	22 (71.0)	38 (64.4)	1.34 (0.53–3.59)	—
Educational level	—	—	—	0.389
Elementary	3 (9.68)	2 (3.33)	1 (Reference)	—
High school	8 (25.8)	13 (21.7)	0.43 (0.04–3.44)	—
Graduate	20 (64.5)	45 (75.0)	0.31 (0.03–2.18)	—
Contact with cats in the penitentiary	—	—	—	0.434
No	13 (48.1)	20 (36.4)	1 (Reference)	—
Yes	14 (51.9)	35 (63.6)	0.62 (0.24–1.60)	—
Meals provided by the penitentiary	—	—	—	0.242
No	12 (40.0)	15 (25.4)	1 (Reference)	—
Yes	18 (60.0)	44 (74.6)	0.52 (0.20–1.34)	—
Consuming of water in the penitentiary	—	—	—	0.988
No	7 (22.6)	12 (20.0)	1 (Reference)	—
Yes	24 (77.4)	48 (80.0)	0.85 (0.30–2.59)	—
Historic of raw meat ingestion	—	—	—	0.593
No	20 (66.7)	35 (58.3)	1 (Reference)	—
Yes	10 (33.3)	25 (41.7)	0.71 (0.27–1.76)	—
Historic of miscarriage	—	—	—	0.365
No	24 (88.9)	43 (79.6)	1 (Reference)	—
Yes	3 (11.1)	11 (20.4)	0.51 (0.10–1.86)	—

## Data Availability

All relevant data are within the manuscript and its supporting information files.
